# Bell’s inequality tests via correlated diffraction of high-dimensional position-entangled two-photon states

**DOI:** 10.1038/s41598-018-23310-9

**Published:** 2018-03-19

**Authors:** Wei Li, Shengmei Zhao

**Affiliations:** 1 Nanjing University of Posts and Telecommunications, Institute of Signal Processing and Transmission, Nanjing, 210003 China; 2 Nanjing University of Posts and Telecommunications, Key Lab Broadband Wireless Communication and Sensor, Network, Ministy of Education, Nanjing, 210003 China; 3 Sunwave Communications CO, Hangzhou, 310053 China

## Abstract

Bell inequality testing, a well-established method to demonstrate quantum non-locality between remote two-partite entangled systems, is playing an important role in the field of quantum information. The extension to high-dimensional entangled systems, using the so-called Bell-CGLMP inequality, points the way in measuring joint probabilities, the kernel block to construct high dimensional Bell inequalities. Here we show that in theory the joint probability of a two-partite system entangled in a Hilbert space can be measured by choosing a set of basis vectors in its dual space that are related by a Fourier transformation. We next propose an experimental scheme to generate a high-dimensional position-entangled two-photon state aided by a combination of a multiple-slit and a 4 f system, and describe a method to test Bell’s inequality using correlated diffraction. Finally, we discuss in detail consequences of such Bell-test violations and experimental requirements.

## Introduction

Entanglement, the superposition of multi-particle product states, is one of the most fascinating properties of quantum mechanics^1,2^. Not only has it enriched our knowledge of fundamental physics, it has also been applied with success in the transmission of quantum information. To date, theoretical research and practical applications have both mainly focused on two-photon polarization entanglement^3–7^ as two-dimensional entanglement (2*D*) is easy to generate and modulate. Researching high-dimensional entanglement has aroused wide research interest^8–10^ because of its critical role in fundamental quantum physics and emergent quantum information technology^11–13^. For instance, higher degrees of freedom enable a single photon to carry more information and thus denser coding^14–17^. Moreover, high-dimensional entangled quantum systems manifest stronger violations of local realism theories than 2*D* systems^18^ and are less vulnerable to environment noise. In terms of security, a quantum-key-distribution protocol based on multi-dimensional entanglement has been proven to be more effective against a symmetric attack^19^. In addition, investigations of high dimensional entanglement would also benefit the understanding of quantum teleportation, a key element in quantum computing^20^.

Conducting quantum information tasks needs the proof of quantum non-locality of the source, which is accomplished through entanglement witnesses such as tests of Bell’s inequality. One of the most famous formulations of Bell’s inequality was developed by Collins, Gisin, Linden, Massar and Popescu (CGLMP) with the objective of describing high-dimensional quantum entangled two-partite systems^21^, it has been successfully applied to various physical systems^8–10^. The operational principle underlying Bell-CGLMP inequality tests can be described as follows. Assume there is a *D*-dimensional quantum entangled two-partite systems shared by the two remotely separated participants, Alice and Bob. Alice and Bob individually take two different measurements (A_1_, A_2_ and B_1_, B_2_) on their respective systems, and then receives *D* possible results for each measurement: A_1_, A_2_ and B_1_, B_2_ = 0, 1, 2, …, *D* − 1. Different measurements are characterized by measurement parameters (*α*_*a*_, *β*_*b*_ with *a*, *b* = 0,1), for example the rotation angle of a polarizer for polarization entanglement^1,22^, the change in computer-generated holograms for orbital angular momentum entanglement^9,23,24^ or the time-delay for time-bin entanglement^25^. With enough measurements, the joint probabilities P(A_*a*_, B_*b*_) can be calculated, and are the building blocks for the construction of the Bell function I_*D*_. If the correlated system can be described by local realism theorems, then I_*D*_ is no larger than 2, whereas if the correlated system is described by quantum theory, then the inequality will be violated. This is the way we test for quantum non-locality.

The development of high-dimensional quantum information has been stimulated by the experimental generation of multidimensional entanglement. The most widely used methods of generating a high-dimensional bi-partite entanglement depend on spontaneous parametric down-conversion (SPDC) by exploring certain degrees of freedom, such as orbital angular momentum^9,24,26^, angular position^23^, momentum^27–29^, position^30–32^, time-bin^25,33^ and frequency^34–36^. These methods actually depend on phase-matching during SPDC^37^, all of which stem from momentum entanglement. A series of impressive work conducted by S. Pádua and collaborators showed that the angular spectrum of the pump beam can be transferred to the joint probability distribution of the down-converted photons^38^, and maximally entangled states can be generated in position space by transmitting the pump beam through a multi-slit array^39,40^. However, in their work, a Bell inequality, which is a physical way to eliminate all classical correlations that could be described by any local realism theorem, has not been tested for these high-dimensional position-entangled two-photon states.

In this paper, based on the experimental configuration describe in Pádua *et al*.^40^, we suggest a 4*f* system to generate a *D*-dimensional position-entangled two-photon state and propose a method to measure the quantum correlations to construct the Bell-CGSLM inequality. Using the Fourier transformation, we show that a high-dimensional two-photon state entangled in single Hilbert space (position space) also has an entanglement behaviour in its dual space (momentum space). The joint probability for the position-entangled two-photon state, which is the elemental building block of Bell-CGLMP inequality tests, can be calculated by taking a set of measurements with basis vectors in momentum space. We show that the violation of Bell-CGMLP inequality can be obtained by detecting the correlated diffraction, which actually is an interference of multiple-slits diffraction, and the influence of the single-slit diffraction can be suppressed by selecting an appropriate ratio between the distance of neighbouring slits and the width of the slit.

## Theoretical scheme

**Figure 1:**
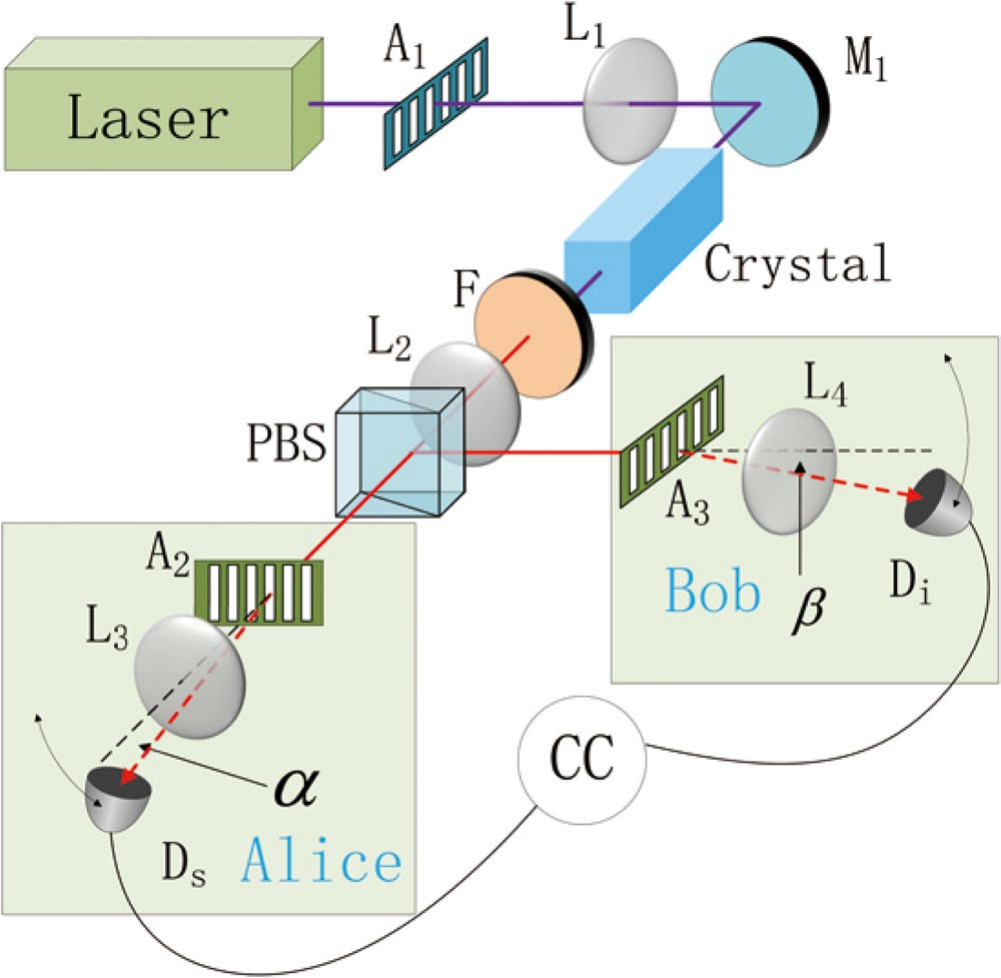
Schematic of the of the optical arrangements for the generation of *D*-dimensional position-entangled two photon state. The plane-wave like pump beam emitted from a quasi-monochromatic laser is transmitted through an opaque plate *A*_1_, a *D*-fold slits, in which the distance between two adjacent slits is *l*, then are divided into *D* even-distributed parts in the transverse plane. A 4*f* optical system comprising lenses *L*_1_ and *L*_2_ focuses the pump beam onto a nonlinear crystal to generate a frequency degenerate type-*II* SPDC, and the transverse plane of the down-converted signal and idler beams are inverted at the output surface of *L*_2_. The remainder pump beam is then filtered by a band-pass filter (F). The signal and idler beams are separated by a polarization beam splitter (PBS) and then transported to slits *A*_1_ and *A*_2_ to undergo diffraction. The diffracted single-photon states are focused by lenses *L*_3_, *L*_4_, and then collected by single-photon detectors *D*_1_ and *D*_2_. The counts are sent to a coincidence circuit to obtain the joint probability.

The schematic diagram of our proposal is shown in Fig. 1. A chromatic plane wave like pump beam is divided into *D* parts in the transverse plane by an opaque plate *A*_1_, composed of D-fold slits separated by a distance *l*. A similar experiment was first realized by Monken *et al*.^38^. The structured pump beam is focused onto a collinear type-*II* nonlinear crystal by lens *L*_1_, and the output beam is then collimated by lens *L*_2_. Here the lenses *L*_1_ and *L*_2_ comprise a 4 *f* system with *A*_1_ lying within the first focal plane of *L*_1_. The co-linearly down-converted photon pairs are then imaged on the second focal plane of *L*_2_ with a same but inverse transverse dimension as the opaque plate *A*_1_. The remaining pump beam is filtered by a band-pass filter *F*. The signal and idler photons with orthogonal polarizations are spatially separated by a polarization beam splitter (PBS), and then directed to the two identical *D*-fold slits *A*_2_ and *A*_3_ possessed by Alice and Bob, respectively. In this proposed experimental setup, *A*_2_ and *A*_3_ locate at the second focal plane of *L*_2_. *A*_1_, *A*_2_ and *A*_3_ have exactly the same number of slits as well as the adjacent slit separation, while the width of each slit in *A*_2_ and *A*_3_ is much smaller then that of *A*_1_. The lenses *L*_3_ and *L*_4_, located behind *A*_2_ and *A*_3_, direct the diffracted photon states to single-photon detectors *D*_1_ and *D*_2_, scanning along the focal planes of *L*_3_ and *L*_4_. The counts from *D*_1_ and *D*_2_ are sent to a coincidence circuit to output the joint probability. Note that, in previous works^38,40^, in order to generate the spatial entanglement, only one focal lens is emplyed in the light path. As a consequence, the transverse dimension of the two-photon state will increase for a longer propagation distance. While our proposed 4*f* imaging system guarantees that the transverse spatial dimension of the down-converted two-photon state remains the same as that of the pump beam, which makes the arrangement of *A*_2_ and *A*_3_ easier. The precise transverse alignment between *A*_2_ and *A*_3_ can be achieved by finding a maximal coincidence count between Alice and Bob.

## Theoretical analysis

Assuming the incoming pump beam is a plane wave with uniform distribution of the amplitude on the transverse plane, the electromagnetic field of the pump beam after the slits *A*_1_ becomes

1 $${{\bf{E}}}_{p}({\bf{r}})=\frac{1}{\sqrt{D}}\sum _{j\mathrm{=0}}^{D-1}\int d{\bf{s}}\cdot {{\bf{E}}}_{p}({{\bf{r}}}_{j}+{\bf{s}})\mathrm{.}$$

Here ${{\bf{E}}}_{p}({{\bf{r}}}_{j}+{\bf{s}})$ is the corresponding electromagnetic field of the pump beam within the *j*-th slit of the plate *A*_1_, **r**_*j*_ the centre position of the *j*-th slit, and ***s*** the position vector along the width direction of the slits ranging from −**d/**2 to **d/**2. As the width of the slit is far larger than the wavelength, the diffraction effect after *A*_1_ is negligible. The lens *L*_1_ introduces a spherical phase to the structured pump beam, leading to different slit states with different momentum, i.e., ${{\bf{E}}}_{p}({\bf{k}})=\frac{1}{\sqrt{D}}{\sum }_{j\mathrm{=0}}^{D-1}\int d{{\bf{q}}}_{p}\cdot {{\bf{E}}}_{p}({{\bf{k}}}_{j}+{{\bf{q}}}_{p})$ with **q**_*p*_ the transverse momentum of the pump beam along the width of the slits.

Very intense incident pump field can be treated classically. Hence, with using the paraxial approximation, the two-photon state generated in degenerate SPDC within the nonlinear crystal can be described as

2 $$|{\rm{\Psi }}\rangle =\frac{1}{\sqrt{D}}\sum _{j\mathrm{=0}}^{D-1}\int d{{\bf{q}}}_{p}\int d{{\bf{q}}}_{s}\int d{{\bf{q}}}_{i}{\hat{a}}_{s}^{+}(\frac{{{\bf{k}}}_{j}}{2}+{{\bf{q}}}_{s}){\hat{a}}_{i}^{+}(\frac{{{\bf{k}}}_{j}}{2}+{{\bf{q}}}_{i}){{\bf{E}}}_{p}({{\bf{k}}}_{j}+{{\bf{q}}}_{p})\delta ({{\bf{q}}}_{p}-{{\bf{q}}}_{s}-{{\bf{q}}}_{i})\delta ({{\bf{q}}}_{s}-{{\bf{q}}}_{i})|0\rangle \mathrm{.}$$

Here ${\hat{a}}_{s}^{+}({{\bf{k}}{\boldsymbol{^{\prime} }}}_{j})$, ${\hat{a}}_{i}^{+}({{\bf{k}}{\boldsymbol{^{\prime} }}}_{j})$ are the creation operators for the signal and idler states of the *j*-th slit. In this equation, the default condition is that the efficiency for SPDC within this momentum range is constant.

After filtering the pump beam, the momentum-entangled down-converted two-photon state at the output surface of the lens *L*_2_ is given by

3 $$|{\rm{\Psi }}\rangle =\frac{1}{\sqrt{D}}\sum _{j\mathrm{=0}}^{D-1}{|j\rangle }_{A}{|j\rangle }_{B}\mathrm{.}$$

With an inverse spherical phase introduced by lens *L*_2_, the momentum-entangled two-photon state now is converted into a *D*-dimensional position-entangled state. Then equation (3) now serves as a position-entangled two-photon state, in which ${|j\rangle }_{A}$ and ${|j\rangle }_{B}$ are the single photon states corresponding to the *j*-th slit, i.e., $\int d{\bf{s}}{|{{\bf{E}}}_{s}({{\bf{r}}}_{j}+{\bf{s}})\rangle }_{A}$ and $\int d{\bf{s}}{|{{\bf{E}}}_{i}({{\bf{r}}}_{j}+{\bf{s}})\rangle }_{B}$, received by Alice and Bob, respectively. Here we emphasize that the entanglement between the transverse position variables of the signal photon and the idler photon from the same slit is negligible; it is determined by the phase-matching condition and the Heisenberg uncertainty principle. Furthermore, note that light paths, for example at the laboratory stage, the crosstalk between neighbouring slit states due to diffraction effect could be reduced by adjusting the width of the slits in *A*_1_.

As any two of these single-slit photon states $|j\rangle$ do not overlap in the transverse plane, all these photon states constitute a complete set of orthogonal basis vectors. The advantage of this optical arrangement is that with the introduction of a 4*f* system in the SPDC process, the spatial extension of the down-converted photon pairs in the transverse plane is the same as the pump beam, which is easily to dealt with by the down-converted two-photon state. Using a finite-dimensional discrete-Fourier-like transformation, the form of equation (3) reads

4 $$\begin{array}{l}|{\rm{\Psi }}\rangle =\frac{1}{\sqrt{D}}\sum _{k\mathrm{=0}}^{D-1}[\sum _{j\mathrm{=0}}^{D-1}\frac{1}{\sqrt{D}}{e}^{i\frac{(k+\alpha )j\cdot 2\pi }{D}}{|j\rangle }_{A}][\sum _{j^{\prime} \mathrm{=0}}^{D-1}\frac{1}{\sqrt{D}}{e}^{-i\frac{(k+\alpha )j^{\prime} \cdot 2\pi }{D}}{|j^{\prime} \rangle }_{B}]=\frac{1}{\sqrt{D}}\sum _{k\mathrm{=0}}^{D-1}{|{\alpha }_{k}\rangle }_{A}{|-{\alpha }_{k}\rangle }_{B}\mathrm{.}\end{array}$$

Here, the states $|j\rangle$ and $|{\alpha }_{k}\rangle$ are connected by a finite-dimensional discrete Fourier transformation, and the integer variables *l*, *k* are the eigenvalues of the state $|j\rangle$ and $|{\alpha }_{k}\rangle$ with respect to certain operators. This transformation plays an important role in quantum entanglement, as it relates time-bin entanglement to frequency entanglement^25,41^, orbital angular momentum entanglement to angular entanglement^9,23^, and momentum entanglement to position entanglement^28,38^.

The transformation in equation (4) also plays a vital role deriving the Bell-CGLMP inequality. The quantum correlation between the *D*-dimensional position-entangled two-photon pairs is detected based on the theoretical work of Collins *et al*.^21^, in which the measurement basis vectors selected by Alice and Bob are

5 $$\begin{array}{ll}{|k\rangle }_{A,a} & =\frac{1}{\sqrt{D}}\sum _{j\mathrm{=0}}^{D-1}{e}^{i\frac{(k+{\alpha }_{a})\cdot j}{D}\cdot 2\pi }{|j\rangle }_{A},\\ {|k^{\prime} \rangle }_{B,b} & =\frac{1}{\sqrt{D}}\sum _{j\mathrm{=0}}^{D-1}{e}^{i\frac{(k^{\prime} +{\beta }_{b})\cdot j}{D}\cdot 2\pi }{|j\rangle }_{B}\mathrm{.}\end{array}$$

Here *α*_*a*_ and *β*_*b*_ are the measurement parameters of Alice and Bob with *a*, *b* = 1, 2 and *α*_1_ = 0, *α*_2_ = 1/2, *β*_1_ = 1/4, *β*_2_ = −1/4. ${|k\rangle }_{A,a}$ and ${|k^{\prime} \rangle }_{B,b}$ are the *k*(*k*′) -th measurement basis vectors of Alice and Bob, *k*(*k*′) = 0, 1, …, *D* − 1. The measurement basis vectors in equation (5) are just those transformed from $|{\alpha }_{k}\rangle$ and $|{\beta }_{k^{\prime} }\rangle$ in equation (4). Accordingly, an inference can be drawn that the correlation of the two-partite systems in the Bell-CGMLP test can be measured in its dual space.

By taking the inner product between the measurement basis vectors in equation (5) and the entangled state in equation (3), we calculate the joint probability *p*(*A*_*a*_ = *k*, *B*_*b*_ = *k*′) in the following form

6 $$p({A}_{a}=k,{B}_{b}=k^{\prime} )=\frac{1}{{D}^{3}}\frac{{\sin }^{2}[\pi (k+{\alpha }_{a}+k^{\prime} +{\beta }_{b})]}{{\sin }^{2}[\frac{\pi }{D}(k+{\alpha }_{a}+k^{\prime} +{\beta }_{b})]}\mathrm{.}$$

As position and momentum are conjugate variables connected by a Fourier transformation, we can assert from equation (4) that for position-entangled two-photon states the measurement of the joint probability of the entanglement can be accomplished by detecting the correlated diffraction. Accounting for single-slit diffraction, each slit state $|j\rangle$ can be written in the momentum representation as

7 $$|j\rangle =\int d\theta A(\theta ){|\theta \rangle }_{j}\mathrm{.}$$

Here, *θ* is the propagation direction for the photon state in momentum space, *λ* the wavelength, and *A*(*θ*) the amplitude for ${|\theta \rangle }_{j}$, which is a sin *c* function of *d* sin *c*(*d* sin *θ*/*λ*) with *d* the width of each slit for *A*_2_ and *A*_3_ (Fig. 1). By substituting equation (7) into equation (3), the joint probability density *p*(*α*, *β*) for detecting a diffracted photon propagating in the direction *α* with respect to the normal of *A*_2_ of Alice’s system and detecting the diffracted photon propagating in the direction of *β* with respect to the normal of *A*_3_ of Bob’s system now reads

8 $$p(\alpha ,\beta )\propto {A}^{2}(\alpha ){A}^{2}(\beta )\cdot \frac{1}{{D}^{3}}\cdot \frac{{\sin }^{{\rm{2}}}[\frac{\pi }{\lambda }lD(\sin \,\alpha +\,\sin \,\beta )]}{{\sin }^{2}[\frac{\pi }{\lambda }l(\sin \,\alpha +\,\sin \,\beta )]}\mathrm{.}$$

Here, *A*(*α*) and *A*(*β*) are the components arising from single-slit diffraction and viewed as scale factors, and *l* is the distance between the centres of two adjacent slits.

There are two main differences between equations (6) and (8): the first is that the joint probability in equation (6) is given for discrete variables whereas that in equation (8) is given for continuous variables, in which case the photon number is not conserved, and the second is that a scale factor *A*^2^(*α*)*A*^2^(*β*) appears in equation (8) that stems from single-slit diffraction. In actual experiments, the joint probability is obtained by counting a sufficient number of photons. Therefore, only the sum of the variables, such as *k* + *α*_*a*_ + *k*′ + *β*_*b*_ and sin *α* + sin *β*, makes sense, making the first difference negligible. The scale factor *A*^2^(*α*)*A*^2^(*β*) influences the maximal value that we obtain for the Bell function *I*_*D*_, and this can be overcome by modulating the ratio between the distance of two adjacent slits *l* and the width of the slit *d*.

Because equations (6) and (8) are equivalent, a set of *α*_*k*,*a*_ and *β*_*k*′,*b*_ can be chosen

9 $$\frac{lD}{\lambda }\,\sin \,{\alpha }_{k,a}=k+{\alpha }_{a},\,\frac{lD}{\lambda }\,\sin \,{\beta }_{k^{\prime} ,b}=k^{\prime} +{\beta }_{b}$$

When *l* is much larger than *λ*, we have on imposing paraxial approximation

10 $${\alpha }_{k,a}=\frac{\lambda }{lD}(k+{\alpha }_{a}),\,{\beta }_{k^{\prime} ,b}=\frac{\lambda }{lD}(k^{\prime} +{\beta }_{b})$$

This means that by setting a correlated diffraction in these angles, we obtain the joint probability to construct the Bell-CGLMP inequality.

**Figure 2:**
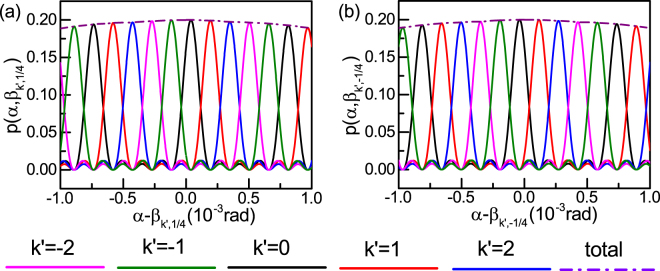
Joint probability *p*(*α*, *β*) of a 5-dimensional position-entangled two-photon state for detecting a diffracted photon in the *α* direction by Alice and a diffracted in the *β* direction by Bob. The diffracted angles for Bob are chosen as $(\frac{1}{4}+k^{\prime} )\frac{\lambda }{lD}$ in (**a**) and $(-\frac{1}{4}+k^{\prime} )\frac{\lambda }{lD}$ in (**b**) where *k*′ = −2, −1, 0, 1, 2. The change in collecting angle for Bob shifts the pattern of the joint probability. The purple dash-dot line is the sum of all the five joint probability curves; the interference pattern appears smeared. With single-slit diffraction, its amplitude decreases as the detection angle increases.

The joint probability *p*(*α*, *β*) for a 5-*D* position-entangled two-photon state (Fig. 2) is obtained by fixing the measurement angle of Bob’s system and scanning that of Alice’s. Here the relevant parameter settings are: the wavelength of signal and idler beam 1550 nm, *l* = 2 mm, and *d* = 0.2 mm. The values of the measurement parameters *β*_*b*_(*b* = 1, 2) chosen by Bob are $\frac{1}{4}$ and $-\frac{1}{4}$, respectively, in Fig. 2(a) and (b), and the five measurement angles for Bob are $\frac{\lambda }{lD}(k^{\prime} \pm \frac{1}{4})$, with *k*′ = −2, −1, 0, 1, 2. From Fig. 2, a change in the measurement angle for Bob shifts the correlated diffraction pattern. This is entirely a correlation phenomenon that has been seen both in quantum^32,42^ and classical correlations^43^. The sum of all five correlated diffraction curves [Fig. 2(a,b); purple dash-dot curve] smears the interference pattern, a phenomena that has also been reported in quantum ghost interference^32,44–46^. From single-slit diffraction, the amplitude of the correlated diffraction tends to decrease as the measurement angle increases.

With the joint probability (Fig. 2), we are going to evaluate Bell’s inequality violations, which enables us to demonstrate quantum non-locality in the 5-*D* position-entangled two-photon state. The scale factor *A*^2^(*α*)*A*^2^(*β*) is determined by the ratio between *l* and *d*, here we have $\frac{l}{d}=10$. By selecting the measurement angle *α* as $\frac{\lambda }{ld}(k+\mathrm{0,}\frac{1}{2})$, *k* = −2, −1, 0, 1, 2, and accounting for single-slit diffraction, the theoretical 5-*D* entanglement Bell function *I*_5_ in this framework is 2.76, which is much larger than 2, the limit predicted by local-hidden-variable theories.

## Discussion

**Figure 3:**
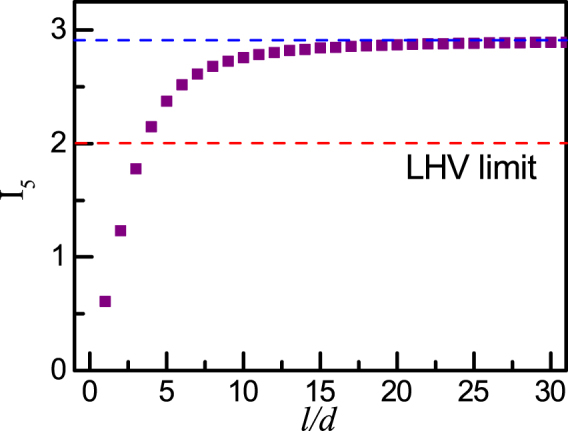
Theoretical Bell function *I*_5_ versus ratio $\frac{l}{d}$ for a 5-dimensional position-entangled two photon state. With $\frac{l}{d}\le 3$, the Bell function *I*_5_ ceases to violate local hidden variable theorems due to single-slit diffraction; with $\frac{l}{d}\ge 10$, *I*_5_ tends to saturate to maximal value 2.91054.

We now discuss the probable influences on the Bell function. From equations (8) and (9), with fixed ratio *l*/*d*, the increase in dimension *D* does affect the fidelity of the Bell function. Hence the only element that may have any influence on the Bell function *I*_*D*_ is the ratio *l*/*d* arising from the single-slit diffraction. Because the spatial entanglement within each slit state can be neglected, the scale factor can be written as the product of the individual coefficient of Alice *A*^2^(*α*) and Bob *A*^2^(*β*). Thus the scale factor only depends on the detection angle. For a relative large *d*, corresponding to a small *l*/*d*, single-slit diffraction dominates. In this case, the scale factor varies significantly within a narrow angular range which leads to a distorted joint probability curve. In contrast, as *d* decreases, the scale factor can be viewed as a constant for a relatively narrow range in detection angle. The measurement results approach the ideal case. From the dependence on *l*/*d* of the theoretical Bell function *I*_5_ for 5 *D* entanglement(Fig. 3), *I*_5_ for $l/d\le 3$ ceases to violate local realism whereas it increases with *l*/*d* for $l/d\ge 10$ and asymptotes to the maximal value of 2.91054.

Moreover, with *l*/*d* = 10, we compared the Bell function *I*_*D*_ in simulations subject to single-slit diffraction with that for ideal cases with entanglement dimension ranging from *D* = 2 to *D* = 8; see Table 1. Here, the Bell function *I*_*D*_ for *l*/*d* → ∞ is adopted as the value for the ideal case when considering the asymptotic behaviour of *I*_*D*_ with respect to *l*/*d* (Fig. 3). This table shows that the Bell function values for *l*/*d* with finite values are less than the maximal value. Furthermore, with *l*/*d* = 10, all values of *I*_*D*_ are larger than 2, which is the limit predicted by local hidden variable theorem. The Bell function values obtained here almost reach 94% of the maximal values for all dimensions. Another interesting point to note is that the Bell function values *I*_*D*_, both simulated and ideal, increase with entanglement dimension *D*, leading to a stronger immunity to environmental noise with higher *D*-dimensional entanglement. This is one attractive reason for pursuing such entanglement^18^.

**Table 1:** Comparison of Bell function values *I*_*D*_ for *l*/*d* = 10 and *l*/*d* = ∞.

*D*	2	3	4	5	6	7	8
*l*/*d* = 10	2.627	2.7221	2.7322	2.759	2.7668	2.782	2.7902
*l*/*d* = ∞	2.8284	2.8729	2.8962	2.9105	2.9244	2.9344	2.9464

In addition to the influence introduced by the single slit diffraction, other factors that might affect the Bell function test include inhomogeneous distribution of the pump field in the transverse plane, less precise alignment between *A*_2_ and *A*_3_ and the angle resolution for single photon detection. The first influence can always be found for a pump beam in the Gaussian mode, where the radial distribution of the light field is described by a Gaussian function. In this case, only a non-maximal entanglement can be generated with a consequently reduced Bell function value. Thus a plane-wave-like pump beam is needed for a larger violation of local realism theorem. The less precise alignment between *A*_2_ and *A*_3_ will lead to a less overlapping between the slit states of the down-converted two photons. However, such influence only causes a reduction in the coincidence counts, leaving the visibility values of the Bell kernel unaffected. The angle resolution of single photon detection Δ*θ* can be written as *s*/*f*, with *s* the width of the single slit attached to the single-photon detectors *D*_*s*_ and *D*_*i*_ on the focal planes of *L*_3_ and *L*_4_ to define the spatial resolution of the detection, *f* the focal lengths of *L*_3_ and *L*_4_. A reasonable operation is to choose Δ*θ* as one-twentieth of the period of the bell function in equation (8), then we have $s=\frac{\lambda f}{10lD}$. By choosing *f* = 1000 *mm* and with the values for the parameters in the above analysis, a slit width of 0.0155 *mm* is derived for precise characterization of the Bell function curve, and this can be achieved in current experimental techniques^40^.

From entanglement generation and Bell-inequality tests, we have shown in this paper that a system entangled in a Hilbert space is also entangled in its Fourier-related dual space. The joint probability for constructing the Bell function can be obtained by choosing the measurement basis vectors in the dual space. The joint probability for a high-dimensional position-entangled two-photon state was also measured in the momentum space. A similar example can be found in high-dimensional orbital angular momentum entangled two-photon states, in which the measurement of the Bell function is taken in the angular position space^9^. In addition, quantum ghost imaging, a method to obtain an image by taking the measurement of its spatial correlation, is actually a measurement of the momentum entanglement in position space^28,31^. In contrast to the assumption that the position of the photon state is a discrete variable, the momentum is a continuous variable in the measurement of the correlated diffraction. In this sense, the photon number is not conserved, and hence leads to reduced detection efficiency. However, this reduction can be compensated by adopting a sufficiently long integral time during single-photon detection.

In conclusion, we present an effective scheme to demonstrate Bell-inequality violation for a high-dimensional position entangled two-photon state. The joint probability, which is the kernel to construct the Bell function for a two-partite system entangled in a Hilbert space, can be measured by choosing a set of basis vectors in its dual space that are related by Fourier transformation. Based on the experimental configuration proposed in Pádua^40^, we introduce a 4*f* system to generate *D*-dimensional position-entangle two-photon states, and then we analysed in detail the measurement scheme implemented for the joint probability, as well as the influence of the single-slit diffraction on the value of the Bell function. We showed that a significant violation of Bell’s inequality is achievable by choosing an appropriate ratio for the distance between two adjacent slits and the width of the slit. The experimental configuration and the Bell inequality test method proposed in this paper can find a niche in performing quantum information tasks such as direct quantum communication^47^ and quantum cryptography^11^ where the requirement of Bell-inequality tests can be realized in momentum space while communications and quantum key distributions are established in position space.
